# Redetermination and absolute configuration of 6-hydroxy­salvinolone

**DOI:** 10.1107/S1600536809053197

**Published:** 2009-12-16

**Authors:** Hoong-Kun Fun, Ching Kheng Quah, Suchada Chantrapromma

**Affiliations:** a X-ray Crystallography Unit, School of Physics, Universiti Sains Malaysia, 11800 USM, Penang, Malaysia; b Crystal Materials Research Unit, Department of Chemistry, Faculty of Science, Prince of Songkla University, Hat-Yai, Songkhla 90112, Thailand

## Abstract

The crystal structure of the title compound [systematic name: 5,6,10-trihydr­oxy-7-isopropyl-1,1,4a-trimethyl-2,3,4,4a-tetra­hydro­phenanthren-9(1*H*)-one], C_20_H_26_O_4_, has been reported previously [Salae *et al.* (2009 ▶). *Acta Cryst.* E**65**, o2379–o2380], but the absolute configuration could not be determined as there was no significant anomalous dispersion using data collected with Mo radiation. The absolute configuration has now been determined by refinement of the Flack parameter with data collected using Cu radiation. The absolute configuration at position 4a of the diterpenoid is (*R*)-methyl; other features of the mol­ecule and its crystal packing are similar to those previously described.

## Related literature

For background to diterpenes, see: Fraga *et al.* (2005 ▶); Hueso-Rodríguez *et al.* (1983 ▶) and Topcu & Ulubelen (1996 ▶). For the previous determination, see: Salae *et al.* (2009 ▶). For bond-length data, see: Allen *et al.* (1987 ▶). For hydrogen-bond motifs, see: Bernstein *et al.* (1995 ▶). For puckering parameters, see: Cremer & Pople (1975 ▶). For the stability of the temperature controller used in the data collection, see Cosier & Glazer, (1986 ▶).
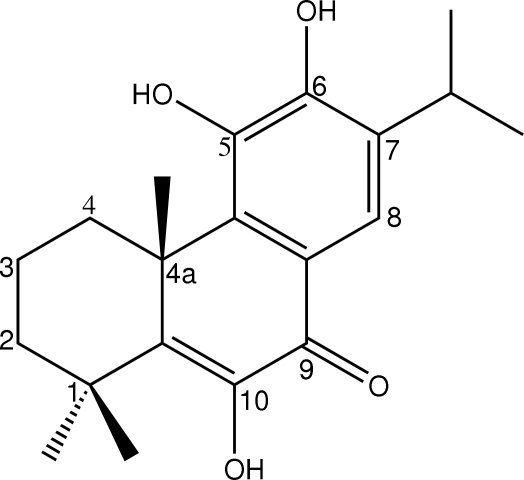


## Experimental

### 

#### Crystal data

C_20_H_26_O_4_
*M* *_r_* = 330.41
Orthorhombic, 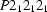

*a* = 9.4908 (1) Å
*b* = 13.1684 (2) Å
*c* = 13.8105 (2) Å
*V* = 1726.02 (4) Å^3^
*Z* = 4
Cu *K*α radiation
μ = 0.70 mm^−1^
*T* = 100 K
0.35 × 0.30 × 0.27 mm

#### Data collection

Bruker APEX Duo CCD area-detector diffractometer
Absorption correction: multi-scan (*SADABS*; Bruker, 2009 ▶) *T*_min_ = 0.790, *T*_max_ = 0.835
6410 measured reflections
2622 independent reflections
2581 reflections with *I* > 2σ(*I*)
*R*_int_ = 0.023

#### Refinement

*R*[*F* ^2^ > 2σ(*F* ^2^)] = 0.033
*wR*(*F* ^2^) = 0.089
*S* = 1.09
2622 reflections
317 parameters
H atoms treated by a mixture of independent and constrained refinement
Δρ_max_ = 0.22 e Å^−3^
Δρ_min_ = −0.22 e Å^−3^
Absolute structure: Flack (1983 ▶), 1609 Friedel pairs
Flack parameter: 0.06 (17)

### 

Data collection: *APEX2* (Bruker, 2009 ▶); cell refinement: *SAINT* (Bruker, 2009 ▶); data reduction: *SAINT*; program(s) used to solve structure: *SHELXTL* (Sheldrick, 2008 ▶); program(s) used to refine structure: *SHELXTL*; molecular graphics: *SHELXTL*; software used to prepare material for publication: *SHELXTL* and *PLATON* (Spek, 2009 ▶).

## Supplementary Material

Crystal structure: contains datablocks global, I. DOI: 10.1107/S1600536809053197/sj2711sup1.cif

Structure factors: contains datablocks I. DOI: 10.1107/S1600536809053197/sj2711Isup2.hkl

Additional supplementary materials:  crystallographic information; 3D view; checkCIF report

## Figures and Tables

**Table 1:** Hydrogen-bond geometry (Å, °)

*D*—H⋯*A*	*D*—H	H⋯*A*	*D*⋯*A*	*D*—H⋯*A*
O1—H1*O*1⋯O2	0.87 (2)	1.93 (2)	2.5607 (14)	128 (2)
O3—H1*O*3⋯O2^i^	0.85 (3)	1.87 (3)	2.6988 (14)	167 (2)
O4—H1*O*4⋯O3	0.81 (3)	2.04 (3)	2.5690 (14)	123 (2)
C14—H14*A*⋯O3^ii^	0.975 (17)	2.542 (17)	3.4774 (17)	160.8 (14)
C15—H15*A*⋯O2^i^	0.98	2.45	3.1933 (17)	132
C18—H18*B*⋯O1	1.005 (18)	2.524 (18)	3.1335 (19)	118.7 (13)
C19—H19*A*⋯O1	0.971 (19)	2.273 (19)	2.903 (2)	121.7 (14)
C20—H20*C*⋯O4	1.01 (2)	2.403 (19)	3.0779 (18)	123.9 (14)

Symmetry codes: (i) 
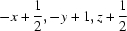
; (ii) 
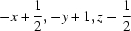
.

**Fig. 1.**
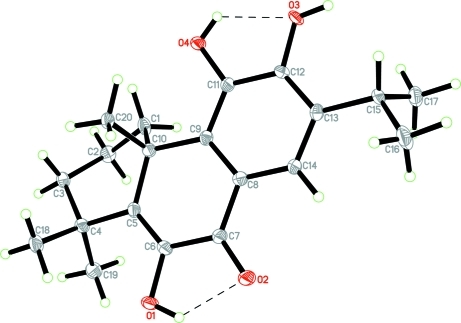
The structure of (I), showing 50% probability displacement ellipsoids and the atom-numbering scheme. Intramolecular hydrogen bonds are shown as dashed lines.

**Fig. 2.**
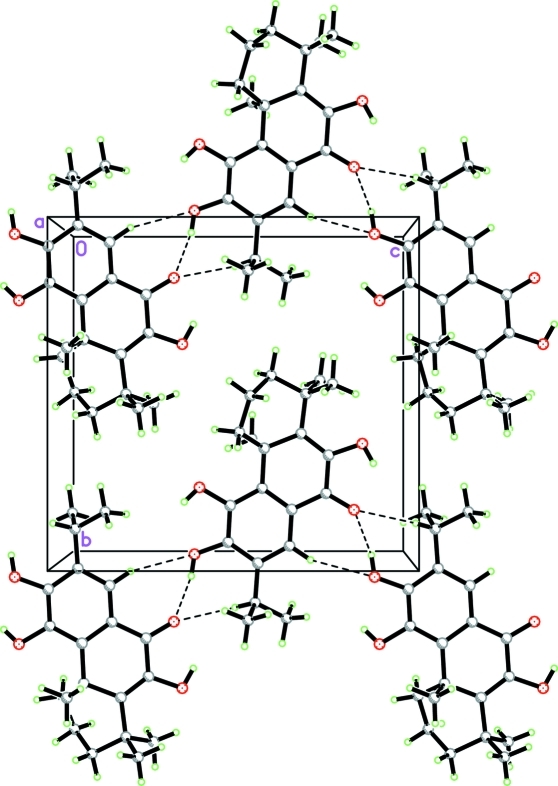
The crystal packing of (I) viewed along the a axis, showing screw chains along the [0 0 1] direction. Hydrogen bonds are shown as dashed lines.

## supplementary crystallographic information

### Comment

The title diterpenoid compound (I) known as 6-hydroxysalvinolone (Topcu & Ulubelen,  1996) or 14-deoxycoleon U (Fraga *et al.*, 2005; Hueso-Rodríguez *et al.*, 1983), was isolated from the roots of *Premna obtusifolia*, a Thai manglove plant which was collected from Satun province in the Southern part of Thailand. Its crystal structure has been reported (Salae *et al.*, 2009) but the absolute configuration could not be determined due to no large anomalous dispersion using a data set collected with Mo radiation. Data on the same sample was recollected using Cu radiation with our newly-installed Bruker Apex-Duo CCD diffractometer and the absolute configuration at atom C10 (or the 4a position) was determined as (*R*)-methyl making use of the large anomalous scattering of Cu Kα*X*-radiation with the Flack parameter being refined to 0.06 (17). We report herein the crystal structure of (I) determined from the Cu data.

Fig. 1 shows the molecular structure of (I); the ring conformations, bond lengths and angles are almost identical to those previously described (Salae *et al.*, 2009).

Fig. 2 shows the crystal packing of (I), intermolecular O—H···O hydrogen bonds and weak C—H···O interactions (Table 1) linked the molecules into infinite one dimensional screw chains along the [0 0 1] direction. This feature and also the O—H···O hydrogen bonds and weak C—H···O interactions are also similar to those in the previous report by Salae *et al.* (2009).

### Experimental

The compound was isolated and the crystal grown as reported by Salae *et al.* (2009).

### Refinement

The H atom attached to C15 was placed in a calculated position with d(C—H) = 0.96 Å and the *U*_iso_ values was constrained to be 1.2*U*_eq_ of the carrier atom. The remaining H atoms were located from the difference map and isotropically refined. The highest residual electron density peak is located at 0.73 Å from C13 and the deepest hole is located at 0.50 Å from O1. 1609 Friedel pairs were used to determine the absolute configuration.

### Crystal data

**Table:**

C_20_H_26_O_4_	*F*(000) = 712
*M**_r_* = 330.41	*D*_x_ = 1.271 Mg m^−^^3^
Orthorhombic, *P*2_1_2_1_2_1_	Cu *K*α radiation, λ = 1.54178 Å
Hall symbol: P 2ac 2ab	Cell parameters from 2622 reflections
*a* = 9.4908 (1) Å	θ = 5.7–62.5°
*b* = 13.1684 (2) Å	µ = 0.70 mm^−^^1^
*c* = 13.8105 (2) Å	*T* = 100 K
*V* = 1726.02 (4) Å^3^	Block, colorless
*Z* = 4	0.35 × 0.30 × 0.27 mm

### Data collection

**Table:**

Bruker APEX Duo CCD area-detector diffractometer	2622 independent reflections
Radiation source: sealed tube	2581 reflections with *I* > 2σ(*I*)
graphite	*R*_int_ = 0.023
φ and ω scans	θ_max_ = 62.5°, θ_min_ = 5.7°
Absorption correction: multi-scan (*SADABS*; Bruker, 2009)	*h* = −10→10
*T*_min_ = 0.790, *T*_max_ = 0.835	*k* = −15→15
6410 measured reflections	*l* = −15→11

### Refinement

**Table:**

Refinement on *F*^2^	Secondary atom site location: difference Fourier map
Least-squares matrix: full	Hydrogen site location: inferred from neighbouring sites
*R*[*F*^2^ > 2σ(*F*^2^)] = 0.033	H atoms treated by a mixture of independent and constrained refinement
*wR*(*F*^2^) = 0.089	*w* = 1/[σ^2^(*F*_o_^2^) + (0.062*P*)^2^ + 0.1924*P*] where *P* = (*F*_o_^2^ + 2*F*_c_^2^)/3
*S* = 1.09	(Δ/σ)_max_ = 0.001
2622 reflections	Δρ_max_ = 0.22 e Å^−^^3^
317 parameters	Δρ_min_ = −0.22 e Å^−^^3^
0 restraints	Absolute structure: Flack (1983), 1609 Friedel pairs
Primary atom site location: structure-invariant direct methods	Flack parameter: 0.06 (17)

### Special details

**Table:**

Experimental. The crystal was placed in the cold stream of an Oxford Cryosystems Cobra open-flow nitrogen cryostat (Cosier & Glazer, 1986) operating at 100.0 (1) K.
Geometry. All esds (except the esd in the dihedral angle between two l.s. planes) are estimated using the full covariance matrix. The cell esds are taken into account individually in the estimation of esds in distances, angles and torsion angles; correlations between esds in cell parameters are only used when they are defined by crystal symmetry. An approximate (isotropic) treatment of cell esds is used for estimating esds involving l.s. planes.
Refinement. Refinement of F^2^ against ALL reflections. The weighted R-factor wR and goodness of fit S are based on F^2^, conventional R-factors R are based on F, with F set to zero for negative F^2^. The threshold expression of F^2^ > 2sigma(F^2^) is used only for calculating R-factors(gt) etc. and is not relevant to the choice of reflections for refinement. R-factors based on F^2^ are statistically about twice as large as those based on F, and R- factors based on ALL data will be even larger.

### Fractional atomic coordinates and isotropic or equivalent isotropic displacement parameters (Å^2^)

**Table:**

	*x*	*y*	*z*	*U*_iso_*/*U*_eq_	
O1	0.26498 (14)	0.14479 (8)	0.64508 (8)	0.0226 (3)	
H1O1	0.266 (3)	0.2024 (19)	0.6146 (17)	0.045 (6)*	
O2	0.25865 (15)	0.33763 (7)	0.66855 (7)	0.0248 (3)	
O3	0.24268 (12)	0.46642 (8)	1.11130 (7)	0.0158 (2)	
H1O3	0.247 (3)	0.530 (2)	1.1204 (17)	0.048 (6)*	
O4	0.25736 (13)	0.27211 (7)	1.09810 (8)	0.0182 (3)	
H1O4	0.257 (3)	0.3146 (19)	1.1402 (19)	0.049 (7)*	
C1	0.14780 (17)	0.11004 (11)	0.98596 (11)	0.0173 (3)	
H1A	0.188 (2)	0.1027 (15)	1.0541 (15)	0.028 (5)*	
H1B	0.068 (2)	0.1522 (15)	0.9886 (15)	0.026 (5)*	
C2	0.09572 (16)	0.00499 (11)	0.95493 (11)	0.0183 (3)	
H2A	0.065 (2)	−0.0370 (17)	1.0113 (16)	0.034 (5)*	
H2B	0.013 (2)	0.0121 (13)	0.9127 (14)	0.023 (4)*	
C3	0.20793 (16)	−0.05336 (11)	0.89923 (11)	0.0161 (3)	
H3A	0.1764 (18)	−0.1257 (14)	0.8862 (12)	0.016 (4)*	
H3B	0.291 (2)	−0.0611 (15)	0.9379 (15)	0.026 (5)*	
C4	0.24229 (18)	−0.00042 (10)	0.80241 (10)	0.0157 (3)	
C5	0.24927 (16)	0.11556 (10)	0.81588 (11)	0.0134 (3)	
C6	0.25717 (17)	0.17835 (11)	0.73906 (10)	0.0166 (3)	
C7	0.25656 (17)	0.28940 (11)	0.74620 (10)	0.0165 (3)	
C8	0.25203 (16)	0.33638 (10)	0.84151 (10)	0.0137 (3)	
C9	0.25196 (14)	0.27429 (10)	0.92394 (10)	0.0125 (3)	
C10	0.26072 (15)	0.15823 (10)	0.91813 (10)	0.0134 (3)	
C11	0.25250 (15)	0.32477 (11)	1.01271 (10)	0.0128 (3)	
C12	0.24735 (15)	0.43145 (11)	1.01817 (9)	0.0127 (3)	
C13	0.24955 (15)	0.49239 (10)	0.93545 (10)	0.0134 (3)	
C14	0.25173 (16)	0.44233 (10)	0.84755 (10)	0.0144 (3)	
H14A	0.257 (2)	0.4837 (13)	0.7891 (12)	0.017 (4)*	
C15	0.24936 (17)	0.60773 (10)	0.94334 (10)	0.0157 (3)	
H15A	0.3062	0.6260	1.0000	0.019*	
C16	0.3152 (2)	0.65897 (12)	0.85566 (14)	0.0298 (4)	
H16A	0.412 (2)	0.6255 (18)	0.8398 (18)	0.050 (7)*	
H16B	0.3240 (19)	0.7331 (16)	0.8679 (15)	0.029 (5)*	
H16C	0.255 (2)	0.6499 (16)	0.8008 (16)	0.036 (5)*	
C17	0.09970 (16)	0.64673 (11)	0.96111 (13)	0.0218 (4)	
H17A	0.059 (2)	0.6171 (15)	1.0234 (16)	0.030 (5)*	
H17B	0.039 (2)	0.6260 (16)	0.9072 (16)	0.034 (5)*	
H17C	0.0999 (18)	0.7216 (14)	0.9708 (13)	0.021 (4)*	
C18	0.38294 (17)	−0.04049 (11)	0.76212 (12)	0.0187 (3)	
H18A	0.461 (2)	−0.0244 (14)	0.8056 (14)	0.023 (4)*	
H18B	0.4045 (19)	−0.0088 (14)	0.6976 (13)	0.021 (4)*	
H18C	0.3732 (19)	−0.1131 (16)	0.7533 (14)	0.024 (5)*	
C19	0.12291 (17)	−0.02847 (12)	0.73087 (12)	0.0210 (4)	
H19A	0.138 (2)	0.0003 (14)	0.6670 (14)	0.026 (5)*	
H19B	0.028 (2)	−0.0059 (14)	0.7535 (15)	0.029 (5)*	
H19C	0.1167 (18)	−0.1038 (14)	0.7267 (13)	0.017 (4)*	
C20	0.41254 (15)	0.13291 (11)	0.95429 (11)	0.0159 (3)	
H20A	0.481 (2)	0.1651 (14)	0.9128 (15)	0.026 (5)*	
H20B	0.4291 (18)	0.0620 (15)	0.9528 (13)	0.021 (4)*	
H20C	0.425 (2)	0.1618 (14)	1.0211 (15)	0.028 (5)*	

### Atomic displacement parameters (Å^2^)

**Table:**

	*U*^11^	*U*^22^	*U*^33^	*U*^12^	*U*^13^	*U*^23^
O1	0.0463 (7)	0.0115 (5)	0.0101 (5)	0.0010 (5)	0.0012 (5)	−0.0010 (4)
O2	0.0506 (7)	0.0120 (5)	0.0117 (5)	0.0003 (5)	0.0000 (5)	0.0024 (4)
O3	0.0281 (6)	0.0087 (5)	0.0106 (5)	−0.0025 (5)	0.0021 (4)	−0.0030 (4)
O4	0.0331 (6)	0.0110 (5)	0.0104 (5)	−0.0001 (5)	−0.0017 (5)	0.0004 (4)
C1	0.0216 (8)	0.0100 (7)	0.0203 (8)	0.0003 (6)	0.0057 (6)	0.0015 (6)
C2	0.0203 (8)	0.0157 (7)	0.0187 (7)	−0.0018 (6)	0.0050 (6)	0.0034 (6)
C3	0.0206 (7)	0.0095 (7)	0.0181 (8)	−0.0006 (5)	−0.0007 (6)	−0.0005 (6)
C4	0.0212 (7)	0.0117 (7)	0.0143 (7)	0.0001 (6)	0.0000 (6)	−0.0013 (5)
C5	0.0139 (7)	0.0109 (7)	0.0154 (7)	0.0012 (6)	−0.0008 (6)	−0.0002 (5)
C6	0.0239 (8)	0.0129 (7)	0.0129 (7)	0.0011 (6)	0.0004 (6)	−0.0022 (5)
C7	0.0248 (8)	0.0121 (7)	0.0125 (7)	−0.0001 (7)	−0.0012 (6)	0.0014 (5)
C8	0.0156 (7)	0.0135 (7)	0.0122 (7)	−0.0001 (6)	−0.0005 (6)	0.0002 (5)
C9	0.0111 (7)	0.0120 (7)	0.0142 (7)	0.0002 (5)	−0.0012 (6)	0.0005 (5)
C10	0.0165 (7)	0.0119 (7)	0.0120 (7)	0.0009 (6)	0.0016 (6)	0.0005 (6)
C11	0.0145 (7)	0.0114 (7)	0.0124 (7)	−0.0001 (6)	0.0004 (6)	0.0013 (5)
C12	0.0143 (7)	0.0130 (7)	0.0109 (7)	−0.0011 (6)	−0.0001 (6)	−0.0021 (5)
C13	0.0136 (6)	0.0110 (7)	0.0156 (7)	−0.0003 (6)	0.0011 (6)	0.0015 (5)
C14	0.0193 (7)	0.0112 (6)	0.0127 (7)	0.0005 (6)	0.0004 (6)	0.0014 (6)
C15	0.0245 (8)	0.0087 (7)	0.0139 (7)	−0.0007 (6)	−0.0013 (6)	−0.0016 (5)
C16	0.0545 (12)	0.0102 (8)	0.0247 (9)	0.0006 (8)	0.0132 (9)	−0.0004 (7)
C17	0.0255 (8)	0.0122 (7)	0.0277 (9)	0.0021 (7)	−0.0027 (7)	−0.0010 (7)
C18	0.0243 (8)	0.0116 (7)	0.0200 (8)	0.0024 (6)	0.0030 (7)	0.0005 (6)
C19	0.0273 (9)	0.0148 (8)	0.0208 (8)	−0.0025 (7)	−0.0050 (7)	−0.0025 (6)
C20	0.0194 (7)	0.0106 (7)	0.0177 (7)	0.0018 (6)	−0.0036 (6)	−0.0010 (6)

### Geometric parameters (Å, °)

**Table:**

O1—C6	1.3732 (17)	C9—C11	1.394 (2)
O1—H1O1	0.87 (3)	C9—C10	1.5327 (18)
O2—C7	1.2465 (18)	C10—C20	1.561 (2)
O3—C12	1.3669 (16)	C11—C12	1.408 (2)
O3—H1O3	0.85 (3)	C12—C13	1.3962 (19)
O4—C11	1.3688 (16)	C13—C14	1.381 (2)
O4—H1O4	0.81 (3)	C13—C15	1.5228 (17)
C1—C2	1.530 (2)	C14—H14A	0.975 (18)
C1—C10	1.558 (2)	C15—C16	1.520 (2)
C1—H1A	1.02 (2)	C15—C17	1.530 (2)
C1—H1B	0.94 (2)	C15—H15A	0.9800
C2—C3	1.522 (2)	C16—H16A	1.04 (2)
C2—H2A	1.00 (2)	C16—H16B	0.99 (2)
C2—H2B	0.99 (2)	C16—H16C	0.96 (2)
C3—C4	1.543 (2)	C17—H17A	1.02 (2)
C3—H3A	1.014 (19)	C17—H17B	0.98 (2)
C3—H3B	0.96 (2)	C17—H17C	0.996 (18)
C4—C18	1.540 (2)	C18—H18A	0.98 (2)
C4—C5	1.5399 (18)	C18—H18B	1.005 (19)
C4—C19	1.548 (2)	C18—H18C	0.97 (2)
C5—C6	1.347 (2)	C19—H19A	0.970 (19)
C5—C10	1.5236 (19)	C19—H19B	1.00 (2)
C6—C7	1.4656 (19)	C19—H19C	0.995 (18)
C7—C8	1.455 (2)	C20—H20A	0.97 (2)
C8—C14	1.3978 (19)	C20—H20B	0.947 (19)
C8—C9	1.4016 (19)	C20—H20C	1.00 (2)
			
C6—O1—H1O1	100.3 (15)	O4—C11—C9	121.07 (12)
C12—O3—H1O3	118.0 (16)	O4—C11—C12	117.42 (12)
C11—O4—H1O4	105.7 (17)	C9—C11—C12	121.51 (12)
C2—C1—C10	114.96 (12)	O3—C12—C13	125.22 (12)
C2—C1—H1A	107.1 (11)	O3—C12—C11	112.81 (12)
C10—C1—H1A	109.4 (11)	C13—C12—C11	121.96 (12)
C2—C1—H1B	106.5 (12)	C14—C13—C12	116.42 (13)
C10—C1—H1B	109.7 (12)	C14—C13—C15	122.60 (13)
H1A—C1—H1B	108.9 (17)	C12—C13—C15	120.98 (12)
C3—C2—C1	111.83 (12)	C13—C14—C8	121.92 (13)
C3—C2—H2A	108.6 (12)	C13—C14—H14A	117.5 (10)
C1—C2—H2A	112.2 (12)	C8—C14—H14A	120.6 (10)
C3—C2—H2B	108.0 (10)	C16—C15—C13	112.65 (12)
C1—C2—H2B	109.8 (11)	C16—C15—C17	111.12 (14)
H2A—C2—H2B	106.2 (16)	C13—C15—C17	110.32 (13)
C2—C3—C4	110.96 (12)	C16—C15—H15A	107.5
C2—C3—H3A	111.0 (10)	C13—C15—H15A	107.5
C4—C3—H3A	109.4 (10)	C17—C15—H15A	107.5
C2—C3—H3B	110.3 (12)	C15—C16—H16A	110.0 (13)
C4—C3—H3B	110.9 (12)	C15—C16—H16B	109.6 (12)
H3A—C3—H3B	104.0 (15)	H16A—C16—H16B	112.1 (16)
C18—C4—C5	110.26 (13)	C15—C16—H16C	109.2 (13)
C18—C4—C3	109.98 (12)	H16A—C16—H16C	108.0 (18)
C5—C4—C3	110.64 (12)	H16B—C16—H16C	107.9 (17)
C18—C4—C19	108.79 (12)	C15—C17—H17A	110.9 (11)
C5—C4—C19	110.20 (12)	C15—C17—H17B	109.4 (12)
C3—C4—C19	106.89 (12)	H17A—C17—H17B	108.1 (16)
C6—C5—C10	119.96 (12)	C15—C17—H17C	110.6 (10)
C6—C5—C4	121.06 (13)	H17A—C17—H17C	105.5 (15)
C10—C5—C4	118.74 (12)	H17B—C17—H17C	112.2 (16)
C5—C6—O1	123.36 (13)	C4—C18—H18A	111.3 (11)
C5—C6—C7	124.01 (13)	C4—C18—H18B	110.8 (10)
O1—C6—C7	112.63 (12)	H18A—C18—H18B	107.4 (15)
O2—C7—C8	124.20 (12)	C4—C18—H18C	107.5 (11)
O2—C7—C6	116.78 (12)	H18A—C18—H18C	111.3 (16)
C8—C7—C6	119.02 (12)	H18B—C18—H18C	108.5 (16)
C14—C8—C9	122.27 (12)	C4—C19—H19A	112.3 (11)
C14—C8—C7	118.58 (12)	C4—C19—H19B	113.0 (12)
C9—C8—C7	119.13 (12)	H19A—C19—H19B	107.5 (16)
C11—C9—C8	115.85 (12)	C4—C19—H19C	108.5 (10)
C11—C9—C10	121.41 (12)	H19A—C19—H19C	110.1 (15)
C8—C9—C10	122.62 (12)	H19B—C19—H19C	105.1 (14)
C5—C10—C9	114.36 (11)	C10—C20—H20A	109.9 (11)
C5—C10—C1	110.97 (12)	C10—C20—H20B	110.9 (11)
C9—C10—C1	109.71 (12)	H20A—C20—H20B	107.8 (15)
C5—C10—C20	106.47 (12)	C10—C20—H20C	108.6 (11)
C9—C10—C20	104.26 (12)	H20A—C20—H20C	107.5 (16)
C1—C10—C20	110.82 (11)	H20B—C20—H20C	112.0 (16)
			
C10—C1—C2—C3	28.97 (17)	C6—C5—C10—C20	−103.23 (16)
C1—C2—C3—C4	−64.94 (16)	C4—C5—C10—C20	71.28 (16)
C2—C3—C4—C18	163.23 (12)	C11—C9—C10—C5	176.24 (12)
C2—C3—C4—C5	41.17 (16)	C8—C9—C10—C5	−7.9 (2)
C2—C3—C4—C19	−78.83 (15)	C11—C9—C10—C1	50.82 (18)
C18—C4—C5—C6	67.99 (19)	C8—C9—C10—C1	−133.35 (13)
C3—C4—C5—C6	−170.13 (14)	C11—C9—C10—C20	−67.90 (16)
C19—C4—C5—C6	−52.1 (2)	C8—C9—C10—C20	107.93 (15)
C18—C4—C5—C10	−106.46 (15)	C2—C1—C10—C5	25.05 (17)
C3—C4—C5—C10	15.42 (19)	C2—C1—C10—C9	152.39 (13)
C19—C4—C5—C10	133.43 (14)	C2—C1—C10—C20	−93.02 (15)
C10—C5—C6—O1	171.82 (14)	C8—C9—C11—O4	−177.74 (13)
C4—C5—C6—O1	−2.6 (2)	C10—C9—C11—O4	−1.6 (2)
C10—C5—C6—C7	−9.0 (2)	C8—C9—C11—C12	2.6 (2)
C4—C5—C6—C7	176.61 (15)	C10—C9—C11—C12	178.69 (13)
C5—C6—C7—O2	−177.13 (14)	O4—C11—C12—O3	−2.3 (2)
O1—C6—C7—O2	2.1 (2)	C9—C11—C12—O3	177.35 (13)
C5—C6—C7—C8	2.2 (3)	O4—C11—C12—C13	176.60 (12)
O1—C6—C7—C8	−178.55 (12)	C9—C11—C12—C13	−3.7 (2)
O2—C7—C8—C14	−0.8 (2)	O3—C12—C13—C14	−178.86 (14)
C6—C7—C8—C14	179.89 (14)	C11—C12—C13—C14	2.3 (2)
O2—C7—C8—C9	−179.14 (16)	O3—C12—C13—C15	1.1 (2)
C6—C7—C8—C9	1.6 (2)	C11—C12—C13—C15	−177.71 (13)
C14—C8—C9—C11	−0.4 (2)	C12—C13—C14—C8	−0.1 (2)
C7—C8—C9—C11	177.83 (14)	C15—C13—C14—C8	179.93 (14)
C14—C8—C9—C10	−176.45 (13)	C9—C8—C14—C13	−0.8 (2)
C7—C8—C9—C10	1.8 (2)	C7—C8—C14—C13	−179.08 (14)
C6—C5—C10—C9	11.3 (2)	C14—C13—C15—C16	−25.5 (2)
C4—C5—C10—C9	−174.14 (12)	C12—C13—C15—C16	154.57 (15)
C6—C5—C10—C1	136.09 (15)	C14—C13—C15—C17	99.31 (17)
C4—C5—C10—C1	−49.39 (18)	C12—C13—C15—C17	−80.62 (18)

### Hydrogen-bond geometry (Å, °)

**Table:**

*D*—H···*A*	*D*—H	H···*A*	*D*···*A*	*D*—H···*A*
O1—H1O1···O2	0.87 (2)	1.93 (2)	2.5607 (14)	128 (2)
O3—H1O3···O2^i^	0.85 (3)	1.87 (3)	2.6988 (14)	167 (2)
O4—H1O4···O3	0.81 (3)	2.04 (3)	2.5690 (14)	123 (2)
C14—H14A···O3^ii^	0.975 (17)	2.542 (17)	3.4774 (17)	160.8 (14)
C15—H15A···O2^i^	0.98	2.45	3.1933 (17)	132
C18—H18B···O1	1.005 (18)	2.524 (18)	3.1335 (19)	118.7 (13)
C19—H19A···O1	0.971 (19)	2.273 (19)	2.903 (2)	121.7 (14)
C20—H20C···O4	1.01 (2)	2.403 (19)	3.0779 (18)	123.9 (14)

Symmetry codes:  (i) −*x*+1/2, −*y*+1, *z*+1/2; (ii) −*x*+1/2, −*y*+1, *z*−1/2.
